# Integrated approach with UHPLC-Q-Exactive-tandem mass spectrometry, network analysis, and molecular docking to determine potential active compounds and mechanisms of Rhizoma Musae decoction in osteoarthritis treatment

**DOI:** 10.3389/fphar.2024.1380335

**Published:** 2025-01-02

**Authors:** Jian Zhang, Wanyan Shen, Fanzhi Liu, Hehe He, Shuquan Han, Lina Luo

**Affiliations:** ^1^ GuiZhou Institute of Subtropical Crops, Guizhou Academy of Agricultural Sciences, Guiyang, China; ^2^ Research and Development Department, Guizhou Weikang Zifan Pharmaceutical Co., Ltd., Guiyang, China

**Keywords:** Rhizoma Musae decoction, UHPLC-Q-Exactive-MS/MS, network analysis, osteoarthritis, signaling pathways

## Abstract

**Objective:**

This study aimed to identify the potential active compounds in Rhizoma Musae decoction and understand their mechanisms of action in osteoarthritis treatment.

**Methods:**

UHPLC-Q-Exactive-MS/MS technology was used for an in-depth analysis of the chemical compounds present in Rhizoma Musae decoction. A network analysis approach was used to construct a comprehensive network of compounds, targets, and pathways, which provided insights into the molecular mechanisms of Rhizoma Musae decoction in osteoarthritis treatment.

**Results:**

The integrated analysis revealed the presence of 534 chemical compounds in Rhizoma Musae decoction, with 7beta-hydroxyrutaecarpine, 7,8-dihydroxycoumarin, pinocembrin diacetate, and scopoletin being identified as potential active compounds. Potential targets such as GAPDH, AKT1, TNF, IL6, and SRC were implicated in key pathways including MAPK signaling pathway, lipid and atherosclerosis, PI3K-Akt signaling pathway, and IL-17 signaling pathway. Molecular docking studies showed significant binding affinity between the core targets and key components. *In vitro* cell experiments have demonstrated that RM decoction can enhance cell proliferation and upregulates the expression of TNFα, IL-6, and SRC, while down-regulating the expression of GAPDH and AKT1.

**Conclusion:**

The potential active compounds present in Rhizoma Musae decoction influence specific targets and signaling pathways involved in osteoarthritis pathogenesis, providing new insights for the functional development and utilization of RM.

## 1 Introduction

Rhizoma Musae (RM), which is known as the dried root or rhizome of *Musa basjoo* Sied.et Zucc. and belongs to the Musa family, has been used as a traditional Chinese herb in Guizhou Province’s Miao region for centuries. The Quality Standards for Traditional Chinese and Ethnic Medicinal Materials in Guizhou Province (Guizhou Medical Products Administration, 2003) indicates that RM has multiple medicinal benefits (Fu et al., 2018), including heat-clearing, detoxifying, thirst-quenching, and diuretic properties. It is widely used to treat conditions such as wind-heat headaches, edema, athlete’s foot, thirst, metrorrhagia, vaginal discharge, erysipelas, and hematospermia. RM is rich in medicinal compounds such as essential oils, phenols, benzophenones, alkaloids, and acenaphthene derivatives, exhibiting a broad spectrum of pharmacological activities, such as anti-inflammatory (Wang et al., 2010), analgesic (Liang et al., 2010), antibacterial (Wei et al., 2010), and antioxidant effects (Liu et al., 2013).

Osteoarthritis (OA), a joint disorder characterized by the progressive degeneration of the articular cartilage (Yao et al., 2023), is on the rise due to factors such as the aging population, increasing obesity rates, and high incidence of traumatic knee injuries. This suggests a forthcoming increase in the occurrence and frequency of OA (Chen et al., 2020). Mechanical damage, inflammation, and aging are significant contributors to the development of osteoarthritis (OA), leading to the gradual deterioration of cartilage (Ouyang et al., 2023). In the pathogenesis of OA, an imbalance of cytokines can trigger the repeated activation of pro-inflammatory cytokines, resulting in damage to internal joint structures such as cartilage (Molnar et al., 2021). Pro-inflammatory cytokines like IL-33, IL-17, IL-6, and IL-22 play crucial roles in various signaling pathways within joints (Englund, 2023). Low-grade inflammation is a notable feature in the early stages of OA (Berenbaum, 2013). The increase in inflammatory factors prompts the breakdown of chondrocyte matrix and contributes to the degeneration of OA chondrocytes. This irreversible process further enhances the release of inflammatory factors, accelerating disease progression.

Network analysis is a multidisciplinary field that integrates systems biology, computer science, and bioinformatics. It employs high-throughput technology to investigate the correlation between diseases and drugs efficiently and economically. The approach aligns with traditional Chinese medicine formulas, known for their attributes targeting multiple levels (Zhao et al., 2023). The Gukang capsule, which primarily contains RM, has shown significant effectiveness in treating osteoporosis, fractures, and injuries (Zhu et al., 2022). The aqueous extract of RM promotes the proliferation, differentiation, and mineralization of bone cells in laboratory settings; upregulates alkaline phosphatase levels; and improves blood rheology, thereby reducing blood viscosity and supporting the fracture healing process (Dong et al., 2019). Despite these findings, a comprehensive understanding of the medicinal components of RM decoction and their effects on OA signaling pathways remains elusive. In this study, UHPLC-Q-Exactive-MS/MS technology is utilized to analyze the chemical constituents of RM decoction. Additionally, network analysis and molecular docking techniques are employed to preliminarily explore the intervention mechanism of RM decoction against OA. These findings establish a robust foundation for further clinical studies and the development of drug formulations involving RM.

## 2 Materials and methods

### 2.1 Experimental instruments

Ultra-high-pressure liquid chromatography was performed using a Vanquish system (Thermo Fisher Scientific, United States). Mass spectrometry analysis was conducted with a Q Exactive HFX Mass Spectrometer (Thermo Fisher Scientific, United States). Centrifugation steps were conducted using Centrifuge 5430 R (Eppendorf, Germany). Mixing was achieved with a SCI-VS vortex mixer (Scilogex, United States), and an ultrasonic cleaner SB25-12DTD (Ningbo Xinzhi Biotechnology Co., Ltd., China) was used for sonication.

### 2.2 Plant materials and chemicals

The RM samples were provided by Guizhou Weikang Zifan Pharmaceutical Co., Ltd., China, with the batch number YC01-003-20230801. Solvents including methanol, acetonitrile, formic acid, isopropanol (all of analytical grade from Anpu), and 95% ethanol (of analytical grade from Shanghai Shenggong) were used. Ultrapure water was utilized throughout all procedures.

### 2.3 Analysis of the chemical composition of RM decoction

#### 2.3.1 Sample preparation

Referred to Gao’s report (Gao et al., 2019), RM decoction was prepared as follows: 10 g of RM samples were powdered and added to a container with 100 mL of ultrapure water. The mixture was then brought to a boil and simmered at low heat, maintaining a slight boil, for 1 h. After cooling for 1 h, 1 mL of the supernatant was mixed with 2 mL of a methanol-acetonitrile solution (1:1, v/v), vortexed for 60 s, and then sonicated at low temperature for 30 min. The mixture was centrifuged at 12,000 rpm for 10 min at 4°C, and the supernatant was collected and chilled at −20°C for 1 h to precipitate the proteins. This process was followed by another centrifugation under the same conditions, after which the supernatant was freeze-dried, resuspended in 100 μL of 50% acetonitrile, vortexed, and centrifuged again at 12,000 rpm for 10 min at 4°C to collect the final supernatant for analysis.

#### 2.3.2 Detection conditions

(1) Chromatographic analysis was performed using a UPLC-Orbitrap-MS system. The UPLC conditions were set with a Waters HSS T3 column (100 × 2.1 mm, 1.8 μm), a column temperature of 40°C, a flow rate of 0.3 mL/min, and an injection volume of 2 μL. The solvent system consisted of water with 0.1% formic acid and acetonitrile with 0.1% formic acid. The gradient program started at 0% B for 1 min, increased to 95% B over 12 min, held at 95% B for 1 min, then returned to 0% B by 17 min.
(2) Mass spectrometric analysis included the use of a Q Exactive HFX Hybrid Quadrupole Orbitrap mass spectrometer with a heated ESI source utilizing the Full-ms-ddMS2 MS acquisition methods. Parameters for the ESI source were set to a spray voltage of −2.8 kV/3.0 kV, sheath gas pressure at 40 arb, aux gas pressure at 10 arb, sweep gas pressure at 0 arb, capillary temperature at 320°C, and aux gas heater temperature at 350°C. The scan range was set from 70 to 1050 Da, with a resolution of 70,000 at the first level and 17,500 at the second level. The compounds were characterized using Sanshu Biotech’s proprietary database, which is specialized in Traditional Chinese Medicine (TCM), along with their unique secondary mass spectrometry fragmentation pattern matching technique. The relative contents of each compound in RM decoction were determined using the area normalization method.

### 2.4 Network analysis research

#### 2.4.1 Obtaining targets related to RM decoction

A search was performed in the NCBI PubChem database (https://pubchem.ncbi.nlm.nih.gov/) to identify compounds and extract their structural information related to RM decoction. Isomeric SMILE structural formulas of compounds associated with RM decoction were inputted into the Swiss Target Prediction Database (http://www.swisstargetprediction.ch/) to identify their targets. To identify the target protein of the selected compound, a screening principle based on a predicted target probability greater than 0 was employed. The UniProt database’s ID mapping tool (https://www.uniprot.org/) was then used to convert UniProt IDs into gene symbols, and after removing duplicates, the targets of the potential active compound were successfully identified.

#### 2.4.2 Identification of OA-related target genes

The keyword “osteoarthritis” was used to identify target genes by extracting relevant data from three disease gene databases: GeneCards (https://www.genecards.org/), Comparative Toxicogenomics Database (CTD) database (https://ctdbase.org/), and DisGeNET database (https://www.disgenet.org/). Genes associated with OA were retrieved from these databases, and the collected information was organized to identify disease targets.

#### 2.4.3 Common targets screening and PPI network construction

The online software program Venny (https://bioinfogp.cnb.csic.es/tools/venny/) was used to predict the common targets between RM decoction and OA. Subsequently, the cross-genes were analyzed using the STRING data analysis platform (http://string-db.org/) to create a protein-protein interaction (PPI) network of common targets, applying a minimum interaction score threshold of ≥0.4. The PPI network diagram and data were saved in TSV format. Cytoscape software (version 3.9.0) was then utilized to visualize the PPI network and construct a multidimensional network depicting the relationship between RM decoction compounds and OA.

#### 2.4.4 Analysis of gene ontology and Kyoto Encyclopedia of Genes and Genomes pathways

In-depth analyses were performed using R software to explore the potential biological functions and key signaling pathways of RM decoction in treating OA. Our comprehensive enrichment analyses included Gene Ontology (GO) and Kyoto Encyclopedia of Genes and Genomes (KEGG) pathways, with a significance criterion set at a q-value of <0.05. The results were organized by descending *p*-values, highlighting significant differences in enrichment.

#### 2.4.5 Molecular docking

The core target-related protein structures (in PDB format) for intersection target screening were obtained from the RCSB database (https://www.rcsb.org/), and the core structure of the potential active compound was sourced from the PubChem database. AutoDock software was used to preprocess the target proteins and small molecule compounds and convert their formats to analyze the protein binding sites. The compounds were then docked using AutoDock Vina 1.1.2 software, with defined docking site coordinates and docking verification. A binding energy value below zero indicates spontaneous binding affinity, with values below −5 kcal·mol^−1^ signifying strong binding activity and those below −7 kcal·mol^−1^ indicating intense binding activity (Hsin et al., 2013).

### 2.5 *In vitro* cell experiments

#### 2.5.1 Cell culture

After resuscitating human osteoblast hFOB1.19 cells (Shanghai Cell Bank, Chinese Academy of Sciences, China), the cells were passaged when they reached 80%–95% confluence. To resuspend the cells, 0.25% Trypsin-EDTA medium (Thermo Fisher, United States), preheated to 37°C, was added. The resuspended cells were then transferred to a new culture dish at a density of 5 × 10^5^ cells/mL and placed in a cell culture incubator set to 37°C with 5% CO_2_.

#### 2.5.2 Cell viability assay

Collect hFOB1.19 cells that have reached the logarithmic phase of growth and treat them with RM decoction at concentrations of 0, 60, 80, 100, 120, 140, and 160 μg/mL, while adding blank culture medium to the control group. The cells should be seeded in a 96-well plate, with three replicates for each group, and each well should contain 3 × 10³ cells. After culturing for 48 h, add 10 μL of CCK-8 reagent to each well and incubate for an additional 1.5 h. Subsequently, use a microplate reader to measure the absorbance (A) at 450 nm, and calculate the cell proliferation rate for each experimental group.

#### 2.5.3 Quantitative real-time polymerase chain reaction(qPCR)

Total RNA was extracted from cells using an TRIzol Reagent (Life Technologies, United States), followed by the synthesis of cDNA (cDNA).The qPCR experiments were conducted using CFX384 Multiple real-time fluorescent quantitative PCR apparatus (Bio-Rad, United States). The PCR amplification conditions were set at 95°C for 30 s, followed by 40 cycles of 95°C for 5 s and 60°C for 30 s. The relative expression level of mRNA was calculated using β-actin as the internal reference gene and the 2^−ΔΔCT^ method. The qPCR primer design was performed using Primer Premier 6.0 and Beacon Designer 7.8 software. The primers were synthesized by Sangon Bioengineering (Shanghai), with the sequences were listed in Table 1.

**TABLE 1:** Primers sequences.

Gene name	Forward primer	Reverse primer
Human β-actin	GAT​GAC​CCA​GAT​CAT​GTT​TGA​GAC	GGA​GTC​CAT​CAC​GAT​GCC​AGT
Human GAPDH	CCA​TGA​CAA​CTT​TGG​TAT​CGT​GGA​A	GGC​CAT​CAC​GCC​ACA​GTT​TC
Human AKT1	GCC​CCA​CTT​CCC​CCA​GTT​CT	CCG​CCT​CTC​CAT​CCC​TCC​AA
Human TNFα	CCA​TGT​TGT​AGC​AAA​CCC​TCA​AGC​T	CCT​TGA​AGA​GGA​CCT​GGG​AGT​AGA​T
Human IL-6	CCT​TCG​GTC​CAG​TTG​CCT​TCT	GTG​TGG​GGC​GGC​TAC​ATC​TTT
Human SRC	CTG​CTT​TGG​CGA​GGT​GTG​GAT​G	CCA​CAG​CAT​ACA​ACT​GCA​CCA​G

#### 2.5.4 Statistical analysis

Data were analyzed using GraphPad Prism version 10.1.2. All results are presented as mean ± SEM. An independent unpaired two-tailed *t*-test was employed to compare the two groups.

## 3 Results

### 3.1 Identification of compounds from RM decoction

**FIGURE 1:**
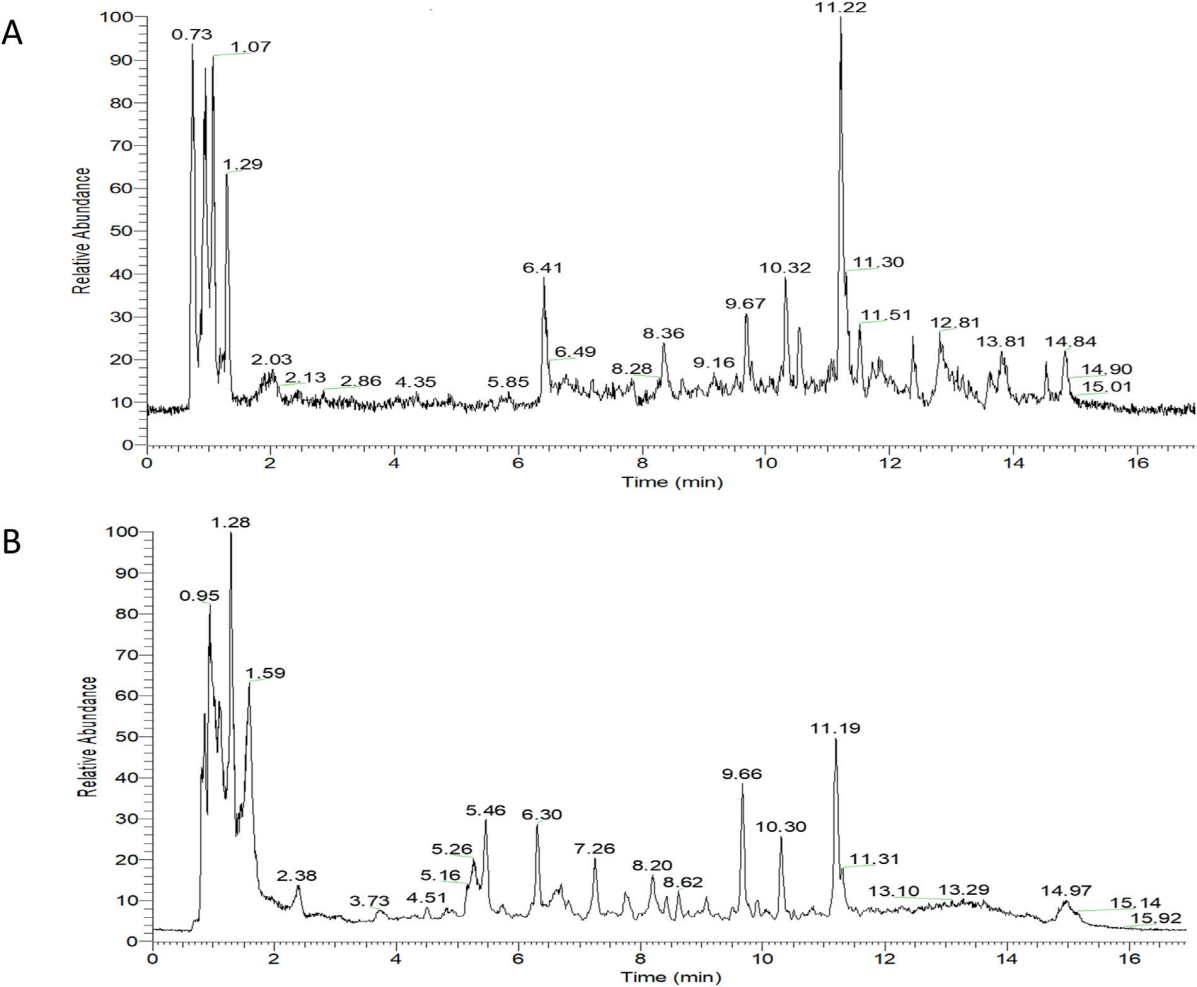
TIC diagram of positive ion **(A)** and negative ion **(B)** modes of compounds from Rhizoma Musae decoction.

Overall, 534 compounds were identified using Sanshu Biotech’s proprietary database, specialized in TCM, and their unique secondary mass spectrometry fragmentation pattern matching technique (Supplementary Table S1). Of these, 299 were detected in the positive ion mode (Figure 1A) and 235 in the negative ion mode (Figure 1B). These compounds were categorized into various groups, including flavonoids (66), prenol lipids (54), organooxygen compounds (50), carboxylic acids and derivatives (Rose-John, 2012), coumarins and derivatives (Lin et al., 2022), fatty acyls (Jiménez-Orozco et al., 2020), benzene and substituted derivatives (Wu et al., 1995), isoflavonoids (Li, 2004), cinnamic acids and derivatives (Gao et al., 2019), phenols (Berenbaum, 2013), benzopyrans (Englund, 2023), anthracenes (Chen et al., 2020), diarylheptanoids (Yao et al., 2023), purine nucleosides (Yao et al., 2023), steroids and steroid derivatives (Yao et al., 2023), and 172 other compounds.

### 3.2 Identification and PPI network analysis of common targets against OA

**FIGURE 2:**
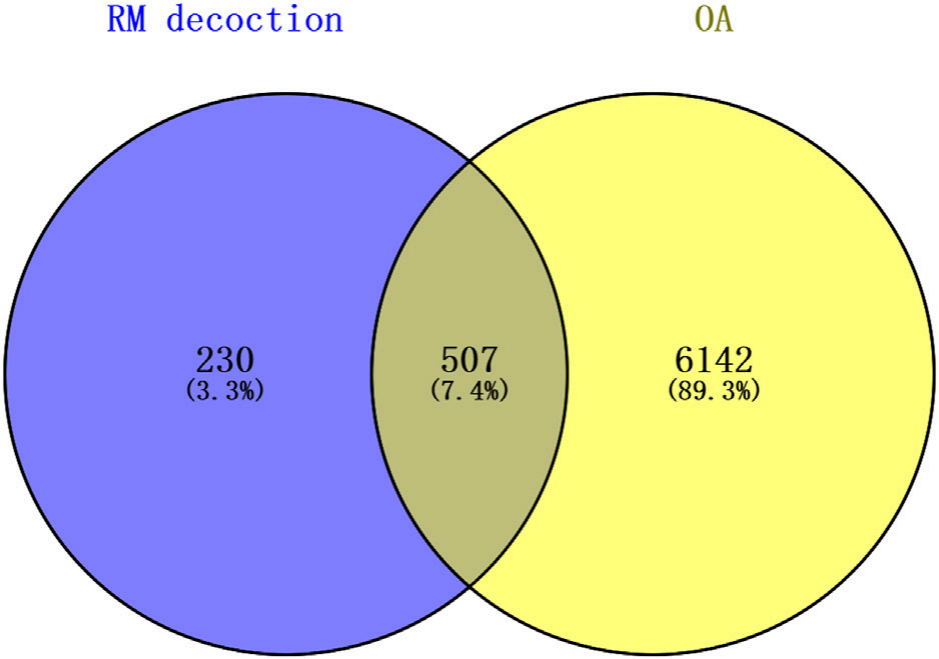
Venn diagram of the common targets between Rhizoma Musae decoction compounds and osteoarthritis. Blue indicates RM decoction and yellow indicates OA.

The top 60 compounds from the RM decoction were selected based on their relative peak areas (Table 2). A total of 737 compound targets were accessed from the Swiss Target Prediction database, and 1174, 6508, and 368 OA-related targets were identified from the GeneCards, CTD, and DisGeNET databases, respectively. Through target gene intersection, the present study identified 507 potential targets for OA treatment (Figure 2).

**TABLE 2:** Relative content of the top 60 compounds in Rhizoma Musae decoction.

No.	Compound	Formula	Rt (min)	m/z	Ion mode	PubChem ID
1	Betaine	C_5_H_11_NO_2_	0.94	118.0864	POS	247
2	3-O-Caffeoylquinic acid	C_16_H_18_O_9_	5.75	353.0874	NEG	1794427
3	Cholinesulfuric acid	C_5_H_13_NO_4_S	0.93	184.0636	POS	485
4	Eleutherazine B	C_22_H_36_N_4_O_6_	7.22	453.2698	POS	20839739
5	ADENOSINE	C_10_H_13_N_5_O_4_	4.06	268.1035	POS	60961
6	7beta-Hydroxyrutaecarpine	C_18_H_12_N_3_O_2_	12.87	303.1010	POS	15225951
7	Ginsenoside Rg1	C_42_H_72_O_14_	10.54	823.4803	POS	441923
8	4-Hydroxybenzoic acid	C_7_H_6_O_3_	6.70	137.0233	NEG	135
9	Trigonelline	C_7_H_7_NO_2_	1.01	138.0549	POS	5570
10	Vanillic acid	C_8_H_8_O_4_	7.24	167.0341	NEG	8468
11	D-mannitol	C_6_H_14_O_6_	0.93	181.0709	NEG	6251
12	Carnitine	C_7_H_15_NO_3_	0.94	162.1123	POS	288
13	2,16-Kauranediol 2-O-beta-D-allopyranoside	C_26_H_44_O_7_	12.43	491.2970	POS	73554066
14	Scopoletin	C_10_H_8_O_4_	8.09	237.0400	NEG	5280460
15	4-Hydroxyphenylpyruvic acid	C_9_H_8_O_4_	5.85	163.0389	POS	979
16	Caffeic acid	C_9_H_8_O_4_	6.80	163.0389	POS	689043
17	Suavioside A	C_26_H_44_O_8_	11.54	507.2922	POS	73821014
18	D-LEUCINE	C_6_H_13_NO_2_	2.01	132.1019	POS	439524
19	Vanillin	C_8_H_8_O_3_	7.76	151.0390	NEG	1,183
20	D-Glutamic acid	C_5_H_9_NO_4_	0.93	148.0603	POS	23327
21	Myo-Inositol	C_6_H_12_O_6_	1.06	179.0552	NEG	892
22	Guanosine	C_10_H_13_N_5_O_5_	2.05	284.0983	POS	135398635
23	D-proline	C_5_H_9_NO_2_	1.03	116.0708	POS	8988
24	Sugeroside	C_26_H_42_O_8_	11.74	505.2764	POS	3082543
25	Opuntiol	C_7_H_8_O_4_	1.06	174.0760	POS	10034839
26	Xylitol	C_5_H_12_O_5_	0.94	151.0601	NEG	6912
27	URIDINE	C_9_H_12_N_2_O_6_	2.79	243.0618	NEG	6029
28	D-Valine	C_5_H_11_NO_2_	0.94	235.1648	POS	71563
29	Gallic acid	C_7_H_6_O_5_	3.11	169.0133	NEG	370
30	7,8-Dihydroxycoumarin	C_9_H_6_O_4_	7.13	223.0243	NEG	5280569
31	Quinic acid	C_7_H_12_O_6_	3.84	173.0446	NEG	6508
32	Lucidone	C_15_H_12_O_4_	9.51	301.0714	NEG	11253859
33	5,7-Dihydroxyphthalide	C_8_H_6_O_4_	6.15	211.0242	NEG	11062751
34	3,4-Dihydroxyphenylacetic acid	C_8_H_8_O_4_	5.09	167.0341	NEG	547
35	D-Arabinose	C_5_H_10_O_5_	0.95	133.0495	POS	854
36	Alpha-Isowighteone	C_20_H_18_O_5_	12.08	303.1009	POS	91885205
37	Aconine	C_25_H_41_NO_9_	12.44	517.3130	POS	20054813
38	Syringolin A	C_24_H_39_N_5_O_6_	12.18	535.3238	POS	42601513
39	N-Isobutyl-2,4,12-octadecatrienamide	C_22_H_39_NO	12.91	378.2750	POS	25221579
40	Stemonidine	C_19_H_29_NO_5_	13.29	332.1863	NEG	24721470
41	[(3,8,12-trihydroxy-24-oxocholan-24-yl)amino]acetate	C_26_H_42_NO_6_^−^	10.54	509.2716	POS	11834768
42	Giffonin R	C_19_H_16_O_3_	10.74	257.0956	POS	134715258
43	4-AMINOBUTYRIC ACID	C_4_H_9_NO_2_	0.94	104.0710	POS	119
44	Linoleic acid	C_18_H_32_O_2_	14.89	313.2729	POS	5280450
45	Paeonilactone A	C_10_H_14_O_4_	6.75	216.1228	POS	10081437
46	3-Hexen-1-ol O-b-D-glucopyranoside	C_12_H_22_O_6_	9.25	261.1340	NEG	5318045
47	D-Glucosamine	C_6_H_13_NO_5_	0.96	180.0864	POS	439213
48	Pierisformoside B	C_26_H_42_O_8_	11.09	505.2763	POS	155978780
49	3′-Hydroxyxanthyletin	C_14_H_12_O_4_	10.30	227.0699	POS	129069558
50	Adenine	C_5_H_5_N_5_	1.30	136.0618	POS	190
51	Preisocalamendiol	C_15_H_24_O	10.54	203.1793	POS	12305706
52	Gamabufotalin	C_24_H_34_O_5_	12.24	403.2450	POS	259803
53	Longicaulenone	C_12_H_18_O_4_	7.91	249.1108	POS	25750965
54	7-Deoxyechinosporin	C_10_H_9_NO_4_	6.96	190.0497	POS	11745823
55	Notoginsenoside R1	C_47_H_80_O_18_	10.22	955.5221	POS	131752529
56	4-Epialyxialactone	C_10_H_16_O_4_	10.08	199.0969	NEG	14194344
57	Pinocembrin diacetate	C_19_H_16_O_6_	9.42	385.0925	NEG	6546286
58	Dehydroacerogenin C	C_19_H_18_O_3_	11.52	259.1113	POS	154790969
59	Excavatin M	C_19_H_20_O_7_	7.81	359.1133	NEG	15871351
60	L (+)-Ascorbic acid	C_6_H_8_O_6_	1.23	221.0297	NEG	54670067

**FIGURE 3:**
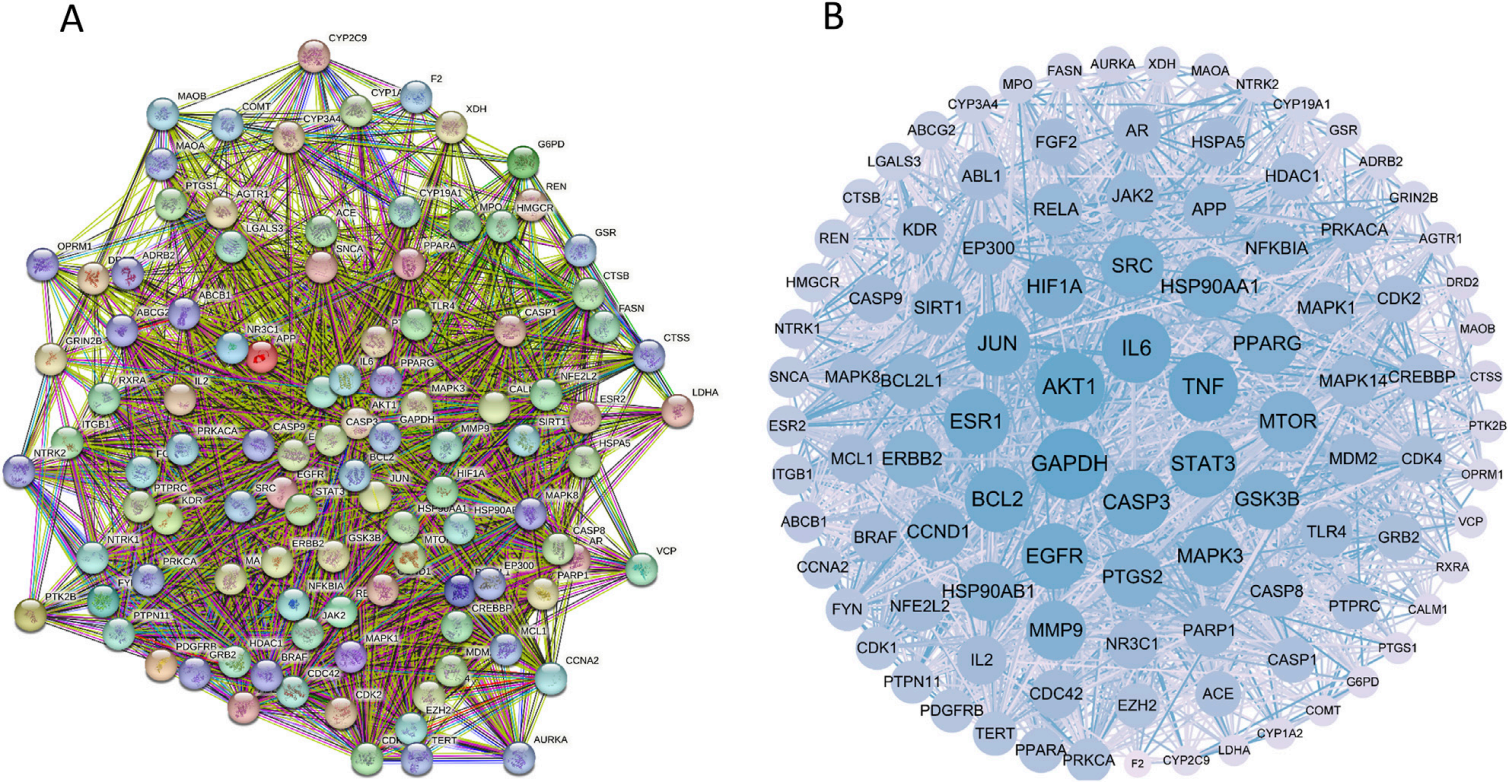
Protein-protein interaction network diagram. **(A)** The PPI network diagram of targets. **(B)** The network topology analysis of the PPI network.

These targets were analyzed using the String database, yielding 507 proteins and 10,739 interaction lines. A drug-disease PPI network diagram was created (Figure 3), highlighting the complex protein relationships. Using Cytoscape software, 103 core targets (Supplementary Table S2) were identified based on the specific criteria of Degree≥42.82936508, betweenness centrality ≥0.002366375, and closeness centrality ≥0.464818506. Among the core targets, GAPDH, AKT1, TNF, IL6, SRC, EGFR, STAT3, HSP90AA1, BCL2, and JUN (Table 3) had higher degrees, suggesting that the compounds present in RM decoction may collectively act on these core targets, leading to its pharmacological effects on OA.

**TABLE 3:** Topological parameter analysis of core targets (top 10).

Targets	Degree	Betweenness centrality	Closeness centrality
GAPDH	269	0.073524	0.680650
AKT1	255	0.049235	0.666225
TNF	244	0.045067	0.655802
IL6	237	0.042281	0.651554
SRC	215	0.055817	0.624845
EGFR	214	0.029535	0.628750
STAT3	211	0.023326	0.627182
HSP90AA1	185	0.021814	0.608222
BCL2	182	0.011213	0.602395
JUN	181	0.018399	0.602395

### 3.3 Elaboration of the “RM decoction-OA” network diagram

**FIGURE 4:**
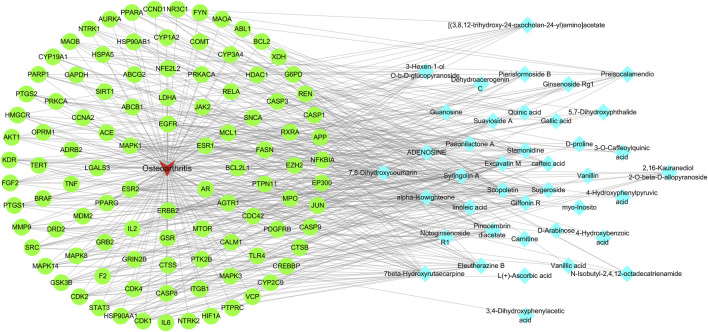
Network of Rhizoma Musae decoction compounds against osteoarthritis. The red inverted triangle represents osteoarthritis, the green circle intersects the therapeutic target, and the blue diamond represents the potential active compound.

A network diagram of the RM decoction against OA was then constructed to further explore the potential association between the core components of RM decoction (Figure 4). The network featured 40 active compound nodes, 103 target nodes, and one disease node. The topological characteristics of this network were analyzed using the Network Analyzer plug-in of Cytoscape software 3.9.0. The potential active compounds with the highest degree values were identified, including 7beta-Hydroxyrutaecarpine (degree = 22), 7,8-dihydroxycoumarin (degree = 21), pinocembrin diacetate (degree = 19), Scopoletin (degree = 19), and Eleutherazine B (degree = 18), among others, detailed in Table 4.

**TABLE 4:** The potential active compounds of Rhizoma Musae decoction against osteoarthritis (top 10).

No.	Compounds	Degree	Betweenness centrality	Closeness centrality	Relative content (%)
1	7beta-Hydroxyrutaecarpine	22	0.023380	0.400560	2.54 ± 1.10
2	7,8-Dihydroxycoumarin	21	0.015359	0.383378	0.68 ± 0.04
3	Pinocembrin diacetate	19	0.016306	0.389646	0.29 ± 0.06
4	Scopoletin	19	0.012880	0.377309	1.87 ± 0.20
5	Eleutherazine B	18	0.011066	0.373368	3.11 ± 0.27
6	Excavatin M	18	0.011991	0.377309	0.29 ± 0.01
7	linoleic acid	18	0.014820	0.383378	0.44 ± 0.08
8	Syringolin A	17	0.012944	0.383378	0.50 ± 0.04
9	Stemonidine	16	0.008540	0.365729	0.48 ± 0.19
10	3-Hexen-1-ol O-b-D-glucopyranoside	15	0.009115	0.375328	0.38 ± 0.04

### 3.4 GO analysis and KEGG pathway enrichment analysis

**FIGURE 5:**
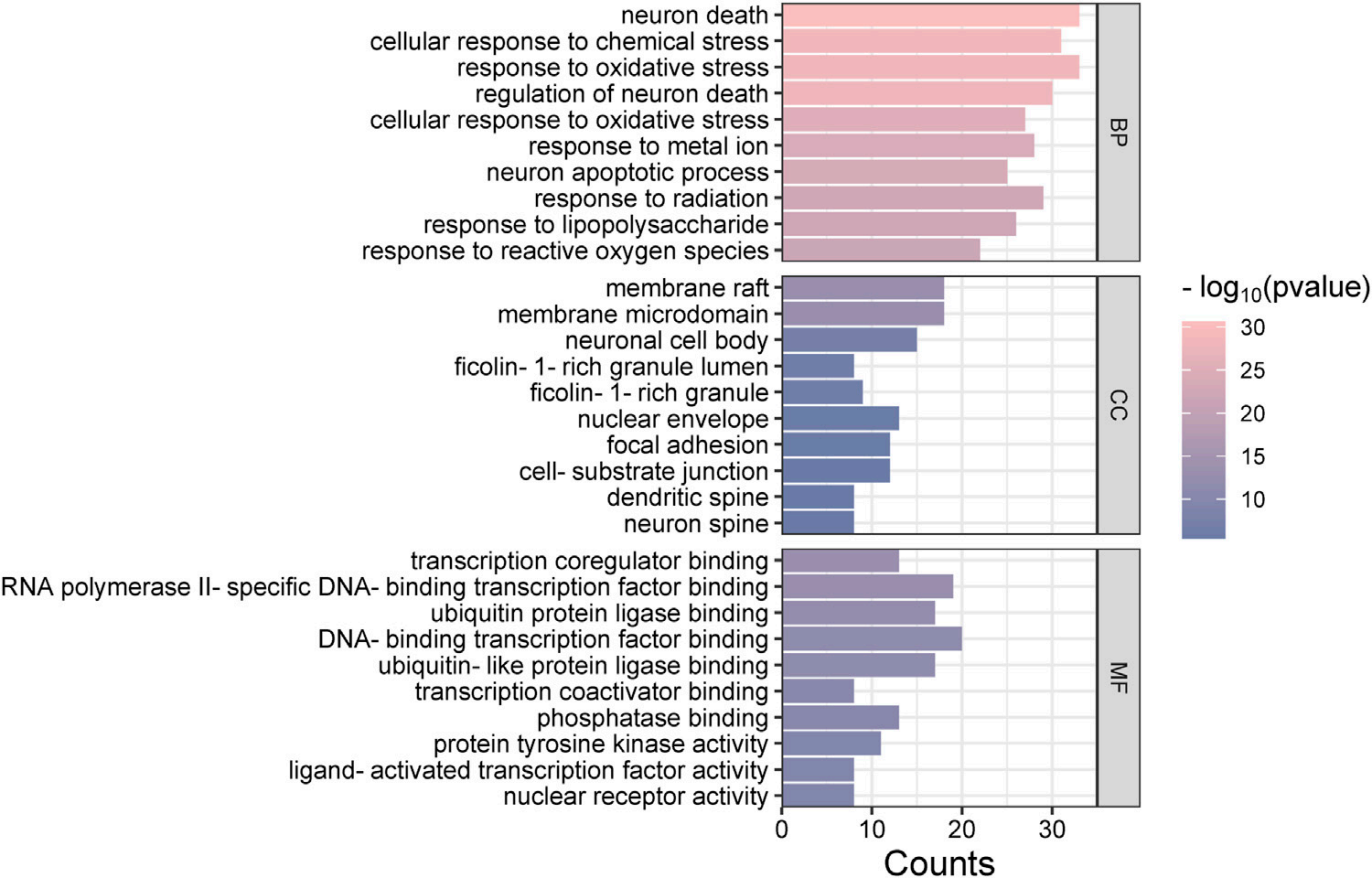
Gene Ontology functional enrichment analysis histogram.

**FIGURE 6:**
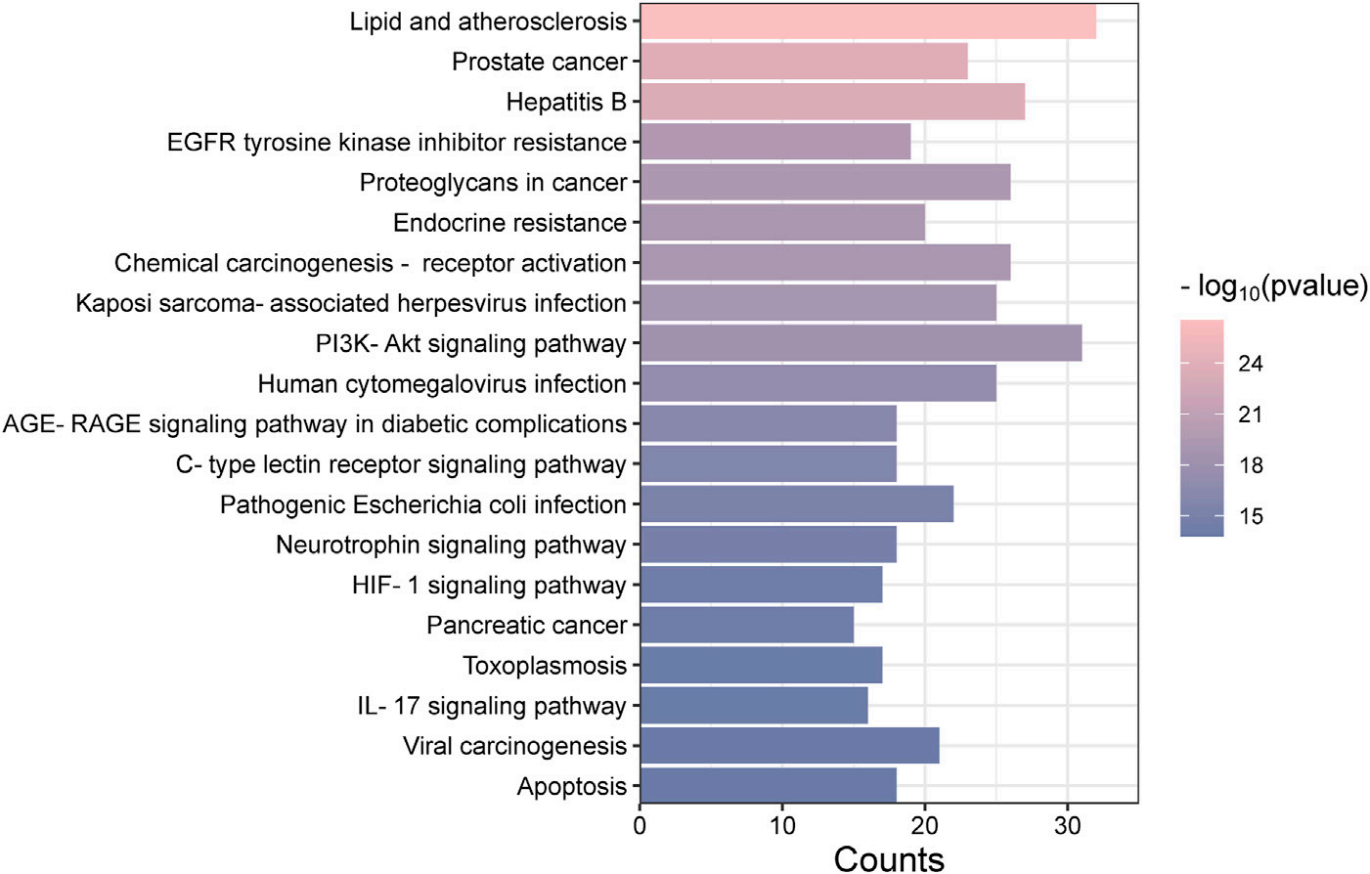
Analysis of Kyoto Encyclopedia of Genes and Genomes pathway enrichment.

**FIGURE 7:**
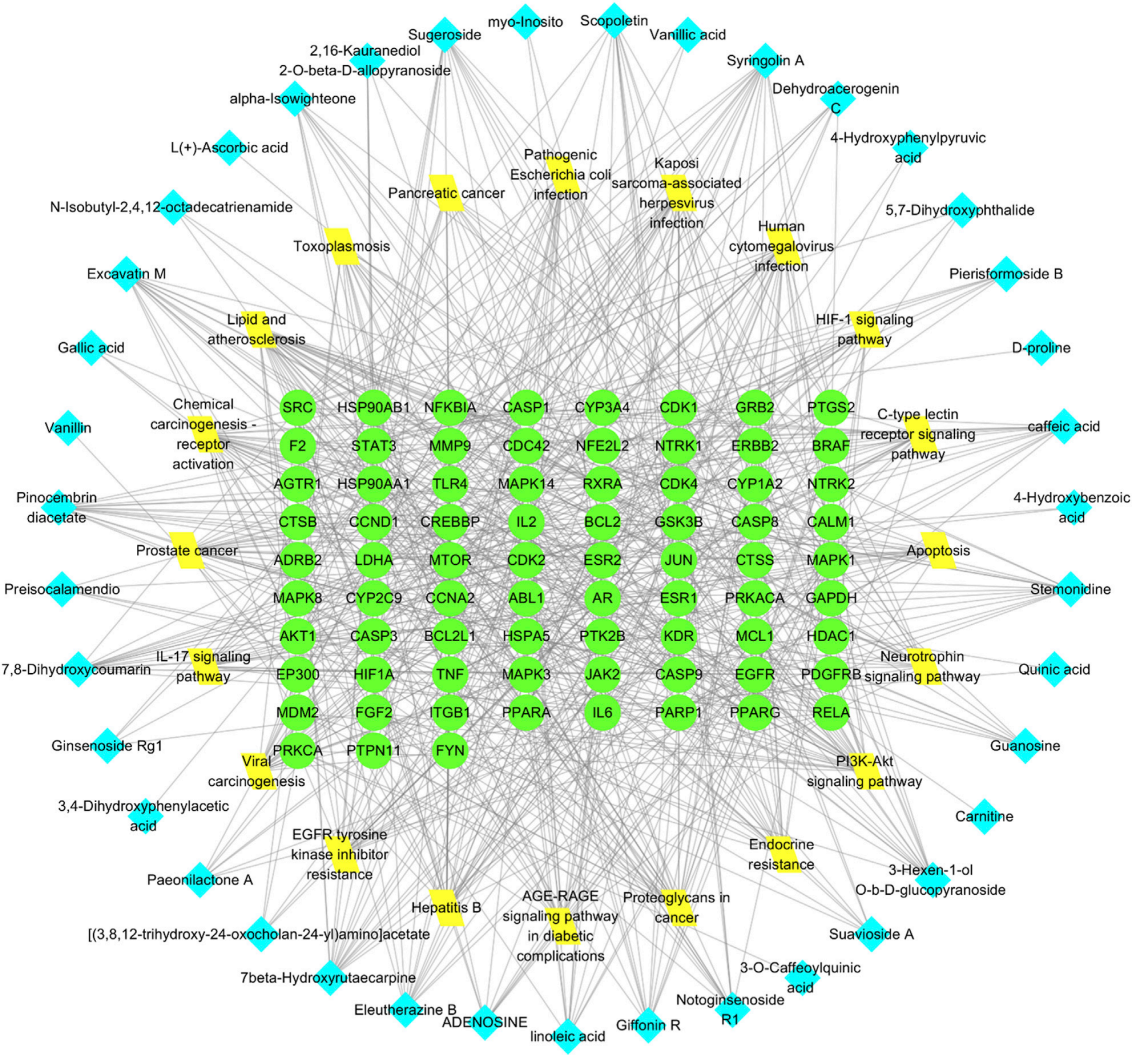
Network of RM decoction component-target-pathway. The green circle represents the target, the yellow diamond represents the pathway, and the blue square represents the drug component.

The “Cluster Profiler” software package for the R language facilitated GO and KEGG enrichment analysis on intersection targets, yielding 2821 entries from the GO analysis (Supplementary Table S3). These included 2536 entries in the Biological Process (BP) category, 182 in the molecular function (MF) category, and 103 in the cellular component (CC) category. Figure 5 presents the top 5 BPs, identified based on the lowest *p*-values: neuron death, cellular response to chemical stress, response to oxidative stress, regulation of neuron death, and cellular response to oxidative stress. The leading MFs were transcription coregulator binding, RNA polymerase II-specific DNA-binding transcription factor binding, ubiquitin protein ligase binding, DNA-binding transcription factor binding, and ubiquitin-like protein ligase binding. The primary CCts included membrane raft, membrane microdomain, neuronal cell body, ficolin-1-rich granule lumen, and ficolin-1-rich granule. KEGG pathway analysis identified 187 signaling pathways (Supplementary Table S4), such as lipid and atherosclerosis, PI3K-Akt signaling pathway, hepatitis B, proteoglycans in cancer, and chemical carcinogenesis–receptor activation, as detailed in Figure 6. An RM decoction component-target-pathway network model was systematically constructed, as displayed in Figure 7.

### 3.5 Molecular docking results

Utilizing Diacerein and corresponding target inhibitors as positive controls, molecular docking was performed between the initial five core components and the first five identified targets, followed by the calculation of binding energy. A binding energy below zero indicates spontaneous binding affinity, with more negative values suggesting greater binding stability. The docking results, illustrated in Table 5, demonstrated spontaneous binding interactions between the core components and the five identified targets. Among the 15 groups, those with binding energy less than −5 kcal·mol^−1^ demonstrated strong binding activity, and two groups with less than −7.0 kcal·mol^−1^ exhibited intense binding activity. Eleutherazine B demonstrates a greater binding affinity towards GAPDH, AKT1, and SRC compared to Diacerein and their respective target inhibitors. Additionally, 7beta-Hydroxyrutaecarpine shows a higher binding capacity to SRC than Diacerein, while Pinocembrin diacetate exhibits a stronger binding affinity to AKT1 than Diacerein.

**TABLE 5:** Predicted binding energies of potential active compounds to core targets combined with positive controls.

Targets	Compounds	
	Name	Binding energy (kcal·mol^−1^)
GAPDH	7,8-Dihydroxycoumarin	−4.84
	7beta-Hydroxyrutaecarpine	−5.79
	Eleutherazine B	−8.36
	Pinocembrin diacetate	−6.64
	Scopoletin	−5.20
	Diacerein^a^	−7.16
	heptelidic acid^b^	−6.44
AKT1	7,8-Dihydroxycoumarin	−4.17
	7beta-Hydroxyrutaecarpine	−4.97
	Eleutherazine B	−6.57
	Pinocembrin diacetate	−5.34
	Scopoletin	−4.16
	Diacerein^a^	−5.09
	BAY 1125976^b^	−5.81
TNF	7,8-Dihydroxycoumarin	−4.60
	7beta-Hydroxyrutaecarpine	−5.44
	Eleutherazine B	−7.04
	Pinocembrin diacetate	−5.85
	Scopoletin	−4.61
	Diacerein^a^	−7.27
	Pomalidomide^b^	−6.00
IL6	7,8-Dihydroxycoumarin	−4.86
	7beta-Hydroxyrutaecarpine	−5.16
	Eleutherazine B	−6.68
	Pinocembrin diacetate	−5.69
	Scopoletin	−5.13
	Diacerein^a^	−6.22
	Curcumin^b^	−6.83
SRC	7,8-Dihydroxycoumarin	−4.40
	7beta-Hydroxyrutaecarpine	−5.20
	Eleutherazine B	−6.69
	Pinocembrin diacetate	−5.08
	Scopoletin	−4.08
	Diacerein^a^	−5.13
	Saracatinib^b^	−6.05

a Positive control drug.

b Target Inhibitors.

**FIGURE 8:**
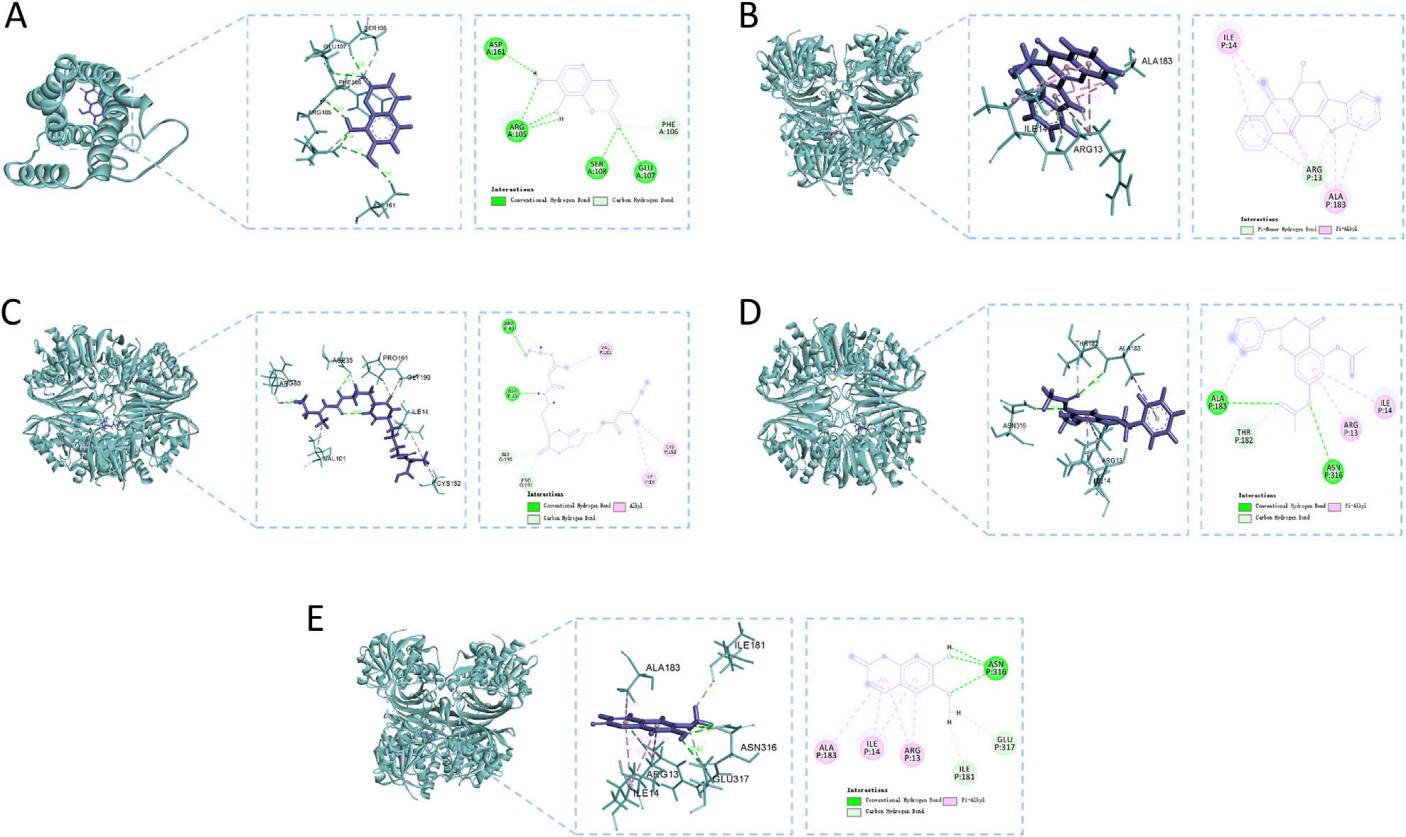
Molecular docking analysis of the main compounds and key targets. **(A)** 7,8-Dihydroxycoumarin - IL6; **(B)** 7beta-Hydroxyrutaecarpine - GAPDH; **(C)** Eleutherazine **(B)** GAPDH; **(D)** Pinocembrin diacetate - GAPDH; **(E)** Scopoletin - GAPDH.

Figure 8 methodically illustrates the visualization of docking conformations with the highest binding energies. 7,8-Dihydroxycoumarin forms a hydrogen bond with IL6 at specific sites, resulting in a binding energy of −4.86 kcal·mol^−1^, indicating robust interaction strength (Figure 8A). The interaction between 7beta-Hydroxyrutaecarpine and GAPDH primarily involves hydrophobic forces, with a binding energy of −5.79 kcal·mol^−1^ (Figure 8B). Eleutherazine B interacts with GAPDH through hydrogen bonds at ARG P:80 and ASP P:35, exhibiting a binding energy of −8.36 kcal·mol^−1^, denoting an intense interaction (Figure 8C). Pinocembrin diacetate forms significant hydrogen bonds with GAPDH at ALA P:183 and ASN P:316 reflected in a binding energy of −6.64 kcal·mol^−1^, indicative of a potent interaction (Figure 8D). Scopoletin engages in hydrogen bonding with GAPDH at ALA P:183 and ASN P:316, displaying a binding energy of −5.20 kcal·mol^−1^, signifying a strong interaction (Figure 8E).

### 3.6 RM decoction promotes the proliferation of hFOB1.19 cells *in vitro*

**FIGURE 9:**
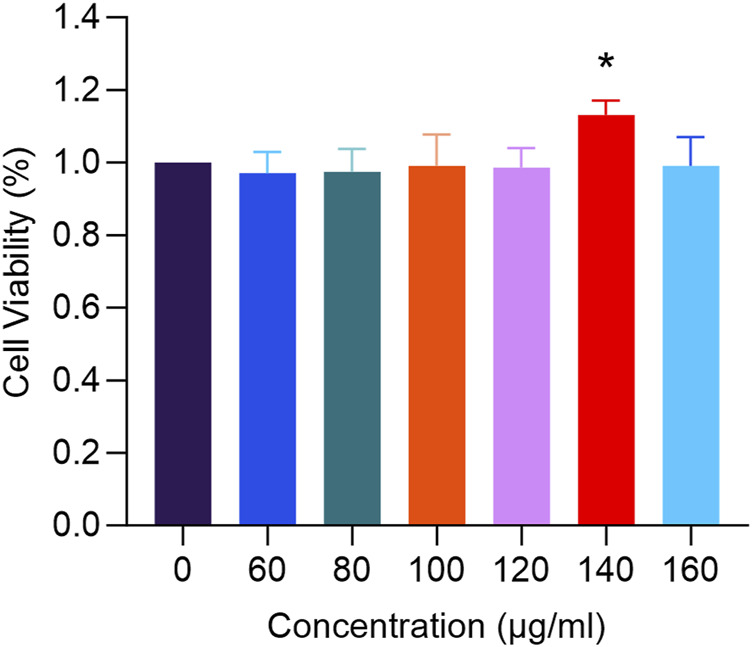
The effects of RM Decoction on the proliferation of hFOB1.19 cells (x ± s, n = 3). * represents *p* < 0.05.

In Figure 9, treatment with RM decoction for 48 h resulted in changes to the proliferation rate of hFOB1.19 cells, which varied with the concentration of the decoction. Notably, at a concentration of 140 μg/mL, the proliferation rate of human osteoblasts was the highest, significantly exceeding that of the control group (*p* < 0.05).

### 3.7 The effects of RM decoction on the expression of related genes in hFOB1.19 cells

**FIGURE 10:**
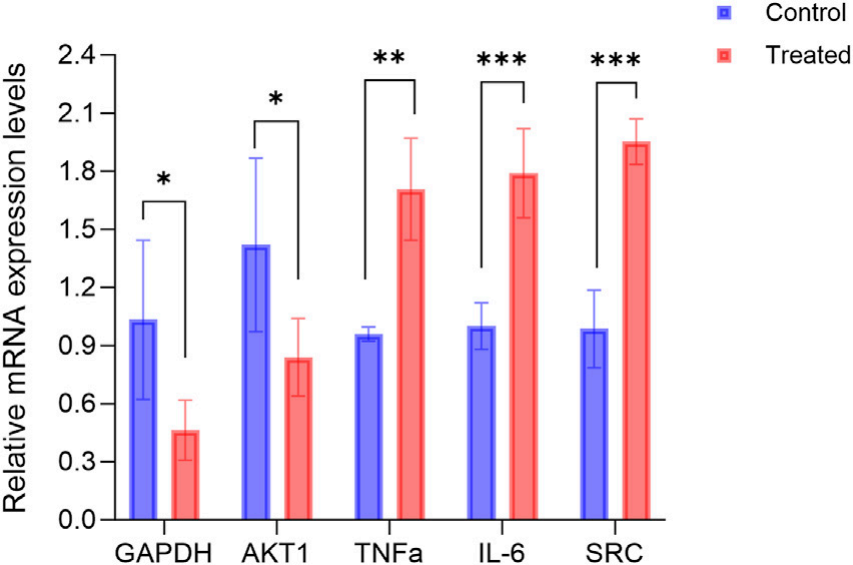
RM decoction regulates the related genes expression in hFOB1.19 Cells. (x ± s, n = 3). * represents *p* < 0.05, ** represents *p* < 0.01, *** represents *p* < 0.001.

Figure 10 illustrates the alterations in gene expression following the treatment of hFOB1.19 cells with 140 μg/mL RM decoction for a duration of 48 h. Notably, RM decoction significantly upregulates the expression of TNFα, IL-6, and SRC, while down-regulating the expression of GAPDH and AKT1.

## 4 Discussion

RM, documented in the Compendium of Materia Medica (Li, 2004), has been utilized in Miao medicine for centuries. This study employs UHPLC-Q-Exactive-MS/MS technology to identify and analyze 534 chemical components present in RM decoction, with a majority being flavonoids, prenol lipids, organooxygen compounds, and others. Furthermore, a component-target correlation analysis on the top 60 chemical components of RM decoction aids in identifying key components for OA treatment.

The decoction method, which involves boiling medicinal materials in water to extract their juices, is a simple form of leaching (Yang and Gao, 2021). This method, considered one of the simplest forms of leaching, is best suited for medicinal materials with active compounds that are soluble in water and can withstand exposure to moisture and heat (Jia et al., 2022). Through mass spectrometry and network analysis, the key components of RM decoction were identified as 7beta-hydroxyrutaecarpine, 7,8-dihydroxycoumarin, Pinocembrin diacetate, and Scopoletin. 7beta-Hydroxyrutaecarpine, derived from evodiacarpine, is a hydroxylation product and a novel indopyridine-quinazoline alkaloid initially discovered in Phellodendron callus tissue (Wu et al., 1995). Experimentally verified, 7beta-Hydroxyrutaecarpine exhibits a range of pharmacological effects, such as analgesic, anti-inflammatory, anti-ulcer, antiemetic, and antidiarrheal properties. (Hu et al., 2018). Another component, 7,8-dihydroxycoumarin, a natural coumarin found in various edible plants of the Thymelaeaceae family, such as Daphne Korean Nakai, Daphne gnidium, Daphne odora, and Daphne oleoides exhibits diverse biological activities, particularly on anti-arthritic (Jiménez-Orozco et al., 2020). Pinocembrin diacetate, a prominent flavonoid, is widely studied for its antimicrobial, anti-inflammatory, antioxidant, and anticancer properties (Rasul et al., 2013). Scopoletin, a naturally occurring coumarin, falls into the category of secondary metabolites and possesses potential anti-inflammatory and anti-tumorigenesis properties (Sakthivel et al., 2022). Diacerein is a slow-acting drug used for osteoarthritis treatment, with anti-inflammatory, antioxidant, and anti-apoptotic properties (Almezgagi et al., 2020). It works by inhibiting the IL-1b system and its downstream signaling pathways (Panova and Jones, 2015). Molecular docking analysis showed potential active compounds in RM decoction, such as 7beta-hydroxyrutaecarpine, 7,8-dihydroxycoumarin, Pinocembrin diacetate, Eleutherazine B, and Scopoletin, effectively binding to the target protein. Organic extraction is a common method for preparing medicinal materials (Lin et al., 2022). Studies have confirmed the anti-inflammatory and analgesic properties of lupenone and β-sitosterol in the ethyl acetate fraction of Rhizoma Musae (Xu et al., 2014). Further research is necessary to explore the potential effectiveness of RM organic extract in osteoarthritis treatment.

The analysis of the PPI network indicates that key proteins such as GAPDH, AKT1, TNF, IL6, and SRC, which have high node Degree values and multiple connections to potential active components, are likely central targets for RM decoction in OA treatment. GAPDH, integral to glycolysis, significantly influences cell proliferation (Sirover, 2021) and apoptosis (Sen et al., 2009). AKT1, a subset of the AKT serine/threonine kinase family encoded by the PKB gene, plays a pivotal role in the PI3K pathway, influencing various downstream effectors. This PI3K/AKT signaling pathway is involved in the abnormal proliferation of bone cells, synovial inflammation, and the formation and differentiation of osteoclasts (Hayer et al., 2009), leading to bone and articular cartilage damage and joint deformity. TNF, which encodes a multifunctional proinflammatory cytokine in the TNF superfamily, is linked to diseases such as autoimmune disorders, insulin resistance, psoriasis, and rheumatoid arthritis (Nie et al., 2013). IL-6, a versatile cytokine, plays roles in immune and nervous system regulation and is associated with antimicrobial molecule production and cytokine activity, as evidenced by research (Rose-John, 2012). SRC, vital in bone resorption by osteoclasts and inhibiting bone formation by osteoblasts (Matsubara et al., 2022), emerges as a potential therapeutic target in osteoporosis treatment. Comparing our results with previous studies suggests that RM decoction could potentially offer anti-inflammatory benefits and promote bone cell proliferation by targeting these specific mechanisms.

The GO analysis covers a range of biological processes, such as neuron death, cellular response to chemical stress, positive regulation of kinase activity, and the positive regulation of the MAPK cascade, among others. The MAPK signaling pathway, critical for various cellular functions including apoptosis, differentiation, and cell proliferation, operates through the activation of nuclear transcription factors (Zheng et al., 2019). The MAPK family, consisting of members such as JNK, ERK, and p38, serves diverse roles. It has been established that ERK1/2 promotes cell proliferation (Chen et al., 2014), while p38 supports cell differentiation. KEGG enrichment analysis has identified the involvement of core targets across 20 signaling pathways, with particular relevance to diseases found in lipid and atherosclerosis, PI3K-Akt signaling pathway, and IL-17 signaling pathway. The lipid and atherosclerosis pathway, for example, involves several inflammation-related targets such as AKT1, TNF, and IL6, underscoring chronic inflammation’s critical role in atherosclerosis progression (Poznyak et al., 2020). This is consistent with literature highlighting atherosclerosis as primarily a chronic inflammatory condition. The PI3K/AKT signaling pathway is pivotal for cell proliferation, differentiation, invasion, and apoptosis (Ma et al., 2014), with 31 core gene targets identified in this study, including AKT1, IL6, and EGFR, among others. These targets are essential components closely associated with the development of inflammatory diseases and cancer progression. IL-17, a highly adaptable cytokine known for its pro-inflammatory capabilities, is linked to genes encoding antimicrobial agents and other cytokines (Tsai et al., 2013). In this research, the IL-17 pathway involves 16 genes, such as TNF, HSP90AA1, and MMP13, significant for host defense, tissue regeneration, and the onset of inflammatory conditions.

## 5 Conclusion

This study aimed to investigate the chemical components and mechanisms of RM decoction in treating OA. The potential components of RM decoction were identified as 7beta-hydroxyrutaecarpine, 7,8-dihydroxycoumarin, Pinocembrin diacetate, and Scopoletin. Network analysis indicates that GAPDH, AKT1, TNF, IL6, and SRC may be important target proteins. These potential active compounds may regulate pathways like the MAPK signaling pathway, Lipid and Atherosclerosis, PI3K-Akt, and IL-17 signaling pathway, potentially offering anti-inflammatory effects and promoting osteocyte proliferation and differentiation. The development of diseases and the pharmacological effects of bioactive substances are complex and constantly changing processes. It is crucial to validate these findings with further experimental and clinical research. Despite the limitations of our study, we have gathered valuable data on the chemical components and mechanism of action of RM in treating OA, offering new perspectives for the functional advancement and utilization of RM.

## Data Availability

The original contributions presented in the study are included in the article/Supplementary material, further inquiries can be directed to the corresponding author.
